# Acetyl-CoA Counteracts the Inhibitory Effect of Antiandrogens on Androgen Receptor Signaling in Prostate Cancer Cells

**DOI:** 10.3390/cancers14235900

**Published:** 2022-11-29

**Authors:** Peter Makhov, Rushaniya Fazliyeva, Antonio Tufano, Robert G. Uzzo, Kathy Q. Cai, Ilya Serebriiskii, Nathaniel W. Snyder, Andrew J. Andrews, Vladimir M. Kolenko

**Affiliations:** 1 Molecular Therapeutics Program, Fox Chase Cancer Center, Philadelphia, PA 19111, USA; 2 Cancer Signaling and Epigenetics Program, Fox Chase Cancer Center, Philadelphia, PA 19111, USA; 3 Urology Unit, Department of Maternal-Child and Urological Sciences, “Sapienza” University of Rome, Viale del Policlinico 155, 00161 Rome, Italy; 4 Department of Surgical Oncology, Fox Chase Cancer Center, Philadelphia, PA 19111, USA; 5 Histopathology Facility, Fox Chase Cancer Center, Philadelphia, PA 19111, USA; 6 Kazan Federal University, 420000 Kazan, Russia; 7 Center for Metabolic Disease Research and the Department of Cardiovascular Sciences, Lewis Katz School of Medicine, Temple University, Philadelphia, PA 19140, USA

**Keywords:** prostate cancer, androgen receptor, enzalutamide, abiraterone, acetyl-coenzyme A

## Abstract

**Simple Summary:**

The androgen receptor (AR) signaling axis is the major therapeutic target in prostate cancer (PC). Second-generation antiandrogens, such as abiraterone acetate and enzalutamide, have shown impressive results in pre- and post-chemotherapy settings, prolonging the survival of PC patients. However, nearly all patients ultimately develop resistance to antiandrogen therapy. Our findings establish the role of the acetyl-CoA-dependent adaptive mechanisms in resistance to antiandrogen therapy.

**Abstract:**

The commonly used therapeutic management of PC involves androgen deprivation therapy (ADT) followed by treatment with AR signaling inhibitors (ARSI). However, nearly all patients develop drug-resistant disease, with a median progression-free survival of less than 2 years in chemotherapy-naïve men. Acetyl-coenzyme A (acetyl-CoA) is a central metabolic signaling molecule with key roles in biosynthetic processes and cancer signaling. In signaling, acetyl-CoA serves as the acetyl donor for acetylation, a critical post-translational modification. Acetylation affects the androgen receptor (AR) both directly and indirectly increasing expression of AR dependent genes. Our studies reveal that PC cells respond to the treatment with ARSI by increasing expression of ATP-citrate lyase (ACLY), a major enzyme responsible for cytosolic acetyl-CoA synthesis, and up-regulation of acetyl-CoA intracellular levels. Inhibition of ACLY results in a significant suppression of ligand-dependent and -independent routes of AR activation. Accordingly, the addition of exogenous acetyl-CoA, or its precursor acetate, augments AR transcriptional activity and diminishes the anti-AR activity of ARSI. Taken together, our findings suggest that PC cells respond to antiandrogens by increasing activity of the acetyl-coA pathway in order to reinstate AR signaling.

## 1. Introduction

Aberrant AR signaling is a key event in PC initiation, progression, and development of castration resistance [1,2,3,4]. Early studies had shown that AR was highly expressed and transcriptionally active in castration-resistant PC (CRPC) [5,6]. Serum levels of prostate-specific antigen (PSA), a well-studied transcriptional target of the AR, invariably rise along with the emergence of castration-resistant disease, indicating that the AR is functionally active in CRPC [3,4,7]. More recent studies show that CRPC cells have increased expression of enzymes mediating androgen synthesis from adrenal steroids and could produce androgens at concentrations that are sufficient to activate AR and promote tumor growth [8,9,10,11]. Therefore, androgen deprivation, as well as inhibition of AR function, have been the mainstay of PC treatment [12,13]. Initially, ADT leads to disease remission and reduction in serum PSA levels. Yet, most of the patients develop metastatic CRPC after a median of 18–36 months [14,15]. Furthermore, about 15 percent of the patients with metastatic disease fail to respond to the therapy [16,17]. Second-generation antiandrogens abiraterone and enzalutamide showed significant survival benefits for patients with CRPC [18]. Abiraterone is a CYP17A1 inhibitor that suppresses the synthesis of androgens. Several recent studies have reported that abiraterone may also directly block AR activity [19,20,21]. Enzalutamide acts by blocking the binding of androgens to AR. Abiraterone and enzalutamide are the most commonly used agents in the first-line treatment of metastatic CRPC. However, nearly all patients develop drug-resistant disease.

Therapeutic failure coincides with different molecular alterations including synthesis of intra-tumoral androgens, upregulation of AR, ligand-independent stimulation of AR, as well as the expression of constitutively active AR variants (AR-Vs) [22,23,24,25,26,27,28,29,30,31]. AR-Vs lack the ligand-binding domain (LBD) but retain the DNA-binding domain and, therefore, are constitutively active in the absence of ligands. Accordingly, antiandrogens are not effective against AR-Vs. [22,23,24,25,26,27,28,29,30,31]. AR-Vs lack the ligand-binding domain (LBD) but retain the DNA-binding domain and, therefore, are constitutively active in the absence of ligands. Accordingly, antiandrogens are not effective against AR-Vs.

Acetyl-coenzyme A (acetyl-CoA) is a central metabolic and signaling molecule that participates in fatty acid synthesis and protein acetylation [32]. The two major enzymes that produce acetyl-CoA are ATP-citrate lyase (ACLY), which generates acetyl-CoA from mitochondria-derived citrate, and acetyl-CoA synthetase 2 (ACSS2), which produces acetyl-CoA from acetate [32,33,34,35,36]. Both ACLY and ACSS2 are aberrantly expressed in PC [37,38,39,40]. Chromatin remodeling via histone acetylation facilitates AR transcriptional activity [41,42,43]. In addition to promoting canonical histone acetylation activity, histone acetyltransferase p300/CBP acetylates the AR [44]. Acetylation of AR enhances coactivator binding, increases its transcriptional activity and promotes PC cell growth [45]. Activation of the Akt/mTOR pathway augments acetyl-CoA production by promoting phosphorylation and activation of ACLY [46]. Akt/mTOR signaling is often constitutively activated in PC cells, mainly through loss of PTEN [47]. Loss of PTEN expression is observed in 27% of primary prostatic tumors and in 79% of CRPC samples [48].

In the present study, we demonstrate, for the first time, that treatment with antiandrogens abiraterone and enzalutamide elevates intracellular levels of acetyl-CoA in PC cells. Correspondingly, the supplementation with exogenous acetyl-CoA, or its precursor acetate, diminishes the anti-AR activity of the ARSI. Critically, inhibition of ACLY, an enzyme responsible for cytosolic acetyl-CoA generation, results in a significant suppression of ligand-dependent and -independent routes of AR activation. Our data suggest that the aberrant acetyl-CoA homeostasis may play a critical role in the development of resistance to therapeutics targeting the AR axis.

## 2. Materials and Methods

### 2.1. The Cells and Culture Conditions

C4-2B human prostate cancer cells were obtained from ATCC (Rockville, MD, USA). LNCaP, 22RV1, and PC-3 human prostate cancer cells were obtained from the Fox Chase Cancer Center Cell Culture facility. Initial stocks were cryopreserved, and at every 6-month interval, a fresh aliquot of frozen cells was used for the experiments. Cells were cultured in RPMI 1640 medium supplemented with 10% fetal bovine serum, gentamicin (50 mg/L), sodium pyruvate (1 mM), and non-essential amino acids (0.1 mM) under conditions indicated in the figure legends.

### 2.2. Antibodies and Reagents

BMS 30314, enzalutamide, abiraterone acetate, acetyl-CoA, sodium acetate, A-485, and C646 were obtained from Cayman Chemical Company (Ann Arbor, MI, USA). Antibodies against AR was obtained from Cell Signaling Technology (Danvers, MA, USA). Antibodies against ACLY and ACSS2 were obtained from ProteinTech (Rosemont, IL, USA). Anti-actin antibodies and R1881 were obtained from MilliporeSigma (Burlington, MA, USA).

### 2.3. Western Blot Analysis

Western blot analysis was performed as described previously [49].

### 2.4. Examination of Intracellular Levels of Acetyl-CoA

Intracellular levels of acetyl-CoA were examined using an acetyl-CoA assay kit (Sigma-Aldrich, St. Louis, MO, USA).

### 2.5. Luciferase Reporter Assay

The luciferase reporter assay was performed as described previously [50]. To generate LNCaP cells with the stable expression of AR-reporter, an AR-Luc cassette was excised from the pGL3-AR-Luc vector and replaced with the CMV promoter of the pLV-CMV-H4-Puro lentiviral vector. Lentiviral infection of LNCap cells was performed by a standard protocol described previously [51].

### 2.6. siRNA Transfection

LNCaP cells were transfected using SMARTpool siRNAs targeting p300 and CBP (Horizon Discovery, Cat# L-003486-00-0005 and L-003477-00-0005, Lafayette, CO, USA) as described in our publication [52].

### 2.7. Examination of PSA and Testosterone Levels

PSA levels in cell culture supernatants were examined using a PSA ELISA kit (Calbiotech). Expression of testosterone in cell lysates and cell culture supernatants was examined using a testosterone ELISA kit (Cayman Chemical, Ann Arbor, MI, USA).

### 2.8. Generation of PC-3 Cells Expressing AR and AR-V7

ORF of the AR was amplified with specific primers using EGFP-AR (plasmid #111215, Addgene, Watertown, MA, USA) as a template; ORF of the AR-V7 splice variant was amplified with specific primers using a AR-V7-pcw107 vector (plasmid #64635, Addgene, Watertown, MA, USA) as a template. Resulting ORFs were re-cloned into EcoRI/BamHI and XbaI/XhoI sites of lentiviral vector pLV-CMV-H4-puro. Lentiviral production, infection of cells, and puromycin selection was performed as described previously [51].

### 2.9. RNA Isolation and qRT-PCR Analysis

Total RNA was isolated using the Mini RNA isolation II Kit (Zymo Research, Irvine, CA, USA) and purified using/via the RNA Clean and Concentrator Kit (Zymo Research, Irvine, CA, USA). Total RNA (1 μg) was reverse transcribed in a final volume of 20 μL with 100 U of Superscript III Reverse Transcriptase (Thermo Fisher Scientific, Waltham, MA, USA) and 50 ng of random hexamer primers according to the manufacturer’s instructions. After reverse transcription, cDNA samples were diluted 40 and 400 times (for genes of interest and 18S rRNA respectively) and 5 μL of diluted cDNA was amplified by real time PCR using PSA (KLK3) PrimeTime primer mix (Hs.PT.58.38546086); and ACLY PrimeTime primer mix (Hs.PT.56a.1709982.g) and ACSS2 PrimeTime primer mix (Hs.PT.58.28278662) (IDT DNA Technologies, Coralville, IA, USA). A custom 18S rRNA Mini qPCR assay (IDT DNA Technologies, Coralville, IA, USA) was used as an internal amplification control. The amplicon was detected with a forward primer 5′-GCTCTTTCTCGATTCCGT, reverse primer 5′-CCAGAGTCTCGTTCGTTATC and probe 5′-TTCTTAGTTG GTGGAGCGATTTGT labeled with 6-FAM and quenched with Jowa-Black FQ. Each sample was run in triplicate for genes of interest in a 20 µL reaction mix using iTaq Universal SYBR Green Supermix (BioRad, Hercules, CA, USA, BioRad, Hercules, CA, USA) and 18S rRNA TaqMan Gene Expression Master Mix according to the manufacturer’s instructions (IDT DNA Technologies, Coralville, IA, USA). Reactions were carried out/performed in an Applied Biosystems 7500 Real-Time PCR System. Analysis of relative genes expression was carried out/performed using the 2−ΔΔCt method.

### 2.10. Immunohistochemistry

Tissues were collected and fixed in 10% phosphate-buffered formaldehyde, dehydrated, and embedded in paraffin. Hematoxylin- and eosin- (H&E) stained sections were used for morphological evaluation purposes. Unstained sections were used for immunohistochemical (IHC) studies. IHC staining was performed on a VENTANA Discovery XT automated staining instrument (Ventana Medical Systems) using VENTANA reagents according to the manufacturer’s instructions. Slides were de-paraffinized using EZ Prep solution (Ventana Medical Systems, #950–102) for 16 min at 72 °C. Epitope retrieval was accomplished with CC1 solution (Ventana Medical Systems, cat #950–224) at 95–100 °C for 32 min. Primary antibodies were tittered with a TBS antibody diluent into user fillable dispensers for use on the automated stainer. Immune complex was detected using the Ventana OmniMap anti-Rabbit detection kit (#760-4311) and developed using the VENTANA ChromMap DAB detection kit (#760-159) according to the manufacturer’s instructions. Slides were counterstained with hematoxylin II (#790-2208) for 8 min, followed by Bluing reagent (#760-2037) for 4 min. The slides were then dehydrated with an ethanol series, cleared in xylene, and mounted. All slides were viewed with a Nikon Eclipse 50i microscope and photomicrographs were taken with an attached Nikon DS-Fi1 camera (Melville, NY, USA).

### 2.11. Statistical Analysis

Statistical analysis was performed using a two-sided Student’s *t*-test. A *p*-value of <0.05 was considered statistically significant.

## 3. Results

### 3.1. Androgen Deprivation Increases Intracellular Levels of Acetyl-CoA

**Figure 1:**
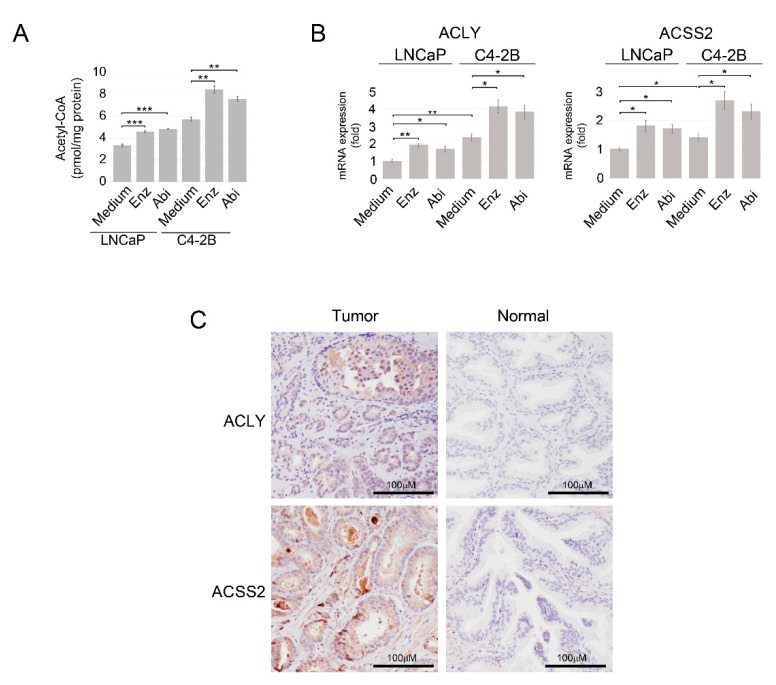
Treatment with enzalutamide and abiraterone increases intracellular levels of acetyl-CoA and promotes expression of ACLY and ACSS2 in PC cells. (**A**) LNCaP and C4-2B cells were cultured in RPMI 1640 medium supplemented with 10% FBS or treated with enzalutamide (Enz) or abiraterone (Abi) (both at 10 μM) for 24 h. Intracellular levels of acetyl-CoA were examined as described in Materials and Methods. (**B**) LNCaP and C4-2B\cells were cultured as described in Panel A. Gene expression was assayed by qRT-PCR. 18S gene was used for normalization. Results are expressed as the mean (*n* = 3) ± SD. * *p* < 0.01; ** *p* < 0.001; *** *p* < 0.0001. (**C**) Representative images of primary moderately differentiated (Gleason score 7) prostate cancer and normal prostate tissue specimens stained for ACLY and ACSS2. The images were derived from a TMA composed of human prostate cancer and normal prostate tissue specimens obtained from patients undergoing treatment at FCCC.

To explore the role of the acetyl-coA pathway in antiandrogen resistance, we examined whether androgen depletion affects intracellular acetyl-CoA levels in PC cells. As demonstrated in Figure 1A, intracellular levels of acetyl-CoA were enhanced in androgen-dependent LNCaP cells and LNCaP-derived metastatic castration-resistant C4-2B cells upon treatment with enzalutamide and abiraterone. The expression of ACLY and ACSS2, the two major acetyl-CoA-producing enzymes, was also enhanced in LNCaP and C4-2B cells treated with antiandrogens (Figure 1B). These data infer that PC cells actively respond to androgen ablation by increasing activity of the nuclear/cytosolic acetyl-coA pathway. Immunohistochemical analysis revealed that ACLY and ACSS2 expression was elevated in PC tissue compared with normal prostate tissue (Figure 1C and Supplementary Figure S1A). Yet, TCGA dataset analysis via the University of Alabama at Birmingham CANcer data analysis Portal (UALCAN) [53] shows that only ACLY mRNA expression is significantly increased in tumor tissue compared with normal tissue (*p* = 2.81 × 10^−6^), whereas ACSS2 mRNA is expressed at reduced levels in tumors samples (*p* = 1.78 × 10^−10^) (Supplementary Figure S1B).

### 3.2. Increase in Acetyl-CoA Levels Compromises the Inhibitory Effect of Abiraterone and Enzalutamide on AR Signaling

**Figure 2:**
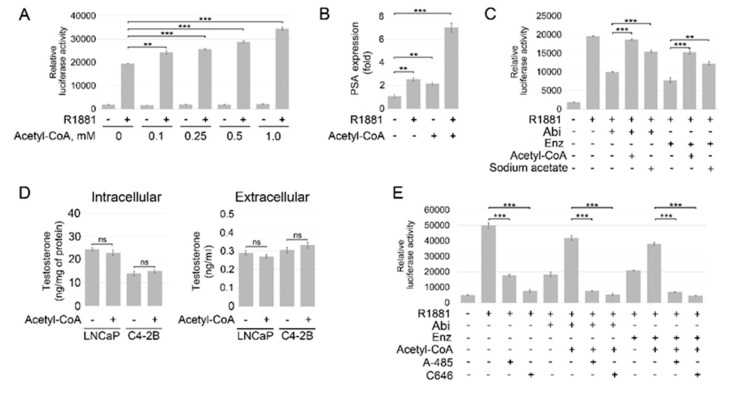
Acetyl-CoA diminishes the inhibitory effect of abiraterone and enzalutamide on AR signaling. (**A**) LNCaP cells were transfected with pGL3-AR-luc and phRL-TK plasmids and cultured under androgen-depleted conditions for 24 h followed by treatment with R1881 (1 nM) and the indicated concentrations of acetyl-CoA for 16 h. Samples were assayed for firefly and renilla luciferase activities using the Dual-Glo Luciferase assay. Values were normalized to Renilla activities. (**B**) LNCaP cells were cultured under androgen-depleted conditions for 24 h followed by treatment with R1881 (1 nM) and acetyl-CoA (0.5 mM) for 6 h. Gene expression was assayed by qRT-PCR. The 18S gene was used for normalization. (**C**) LNCaP cells were transfected with pGL3-AR-luc and phRL-TK plasmids and cultured under androgen-depleted conditions for 24 h followed by treatment with enzalutamide (Enz) (1 μM), abiraterone acetate (5 μM) (Abi), R1881 (1 nM), sodium acetate (10 mM), and acetyl-CoA (0.1 mM) for 16 h. (**D**) LNCaP and C4-2B PC cells were cultured under androgen-depleted conditions for 24 h followed by treatment with acetyl-CoA (0.1 mM) for 48 h. Expression of testosterone in cell lysates and cell culture supernatants was examined using a testosterone ELISA kit (Cayman Chemical). (**E**) Aliquots of LNCaP cells described in Panel C were pre-treated with or without p300/CBP inhibitors A-485 (1 μM) or C646 (25 μM). Results are expressed as the mean (*n* = 3) ± SD. ** *p* < 0.001; *** *p* < 0.0001; ns—non-significant.

**Figure 3:**
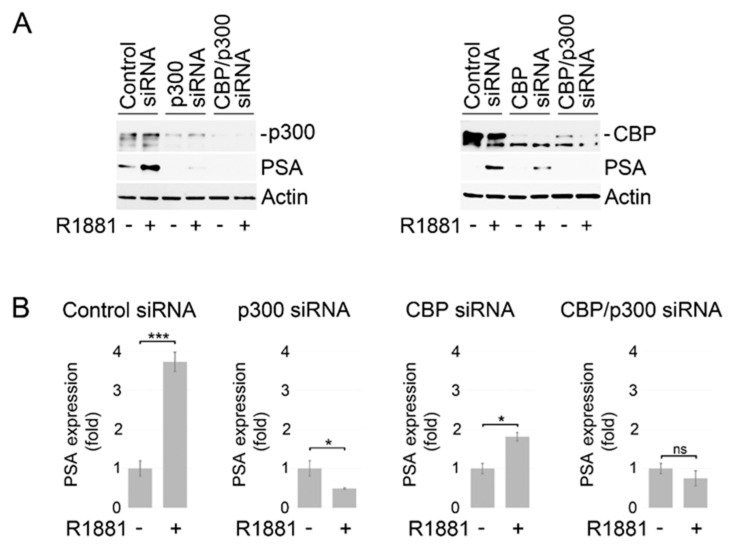
Knockdown of p300 and CBP inhibits AR signaling. (**A**) Analysis of p300, CBP, and PSA protein expression in LNCaP cells carrying the separate or combined p300 and CBP knockdowns. Forty-eight hours after siRNA transfection, LNCaP cells were placed in medium supplemented with charcoal-stripped serum for 24 h followed by stimulation with R1881 (1 nM) for 18 h. Protein expression was examined using Western blot analysis. (**B**) PSA gene expression was examined in LNCaP cells carrying the separate or combined p300 and CBP knockdowns. Forty-eight hours after siRNA transfection, LNCaP cells were placed in medium supplemented with charcoal-stripped serum for 24 h followed by stimulation with R1881 (1nM) for 6 h. Gene expression was assayed by qRT-PCR. 18S gene was used for normalization. Results are expressed as the mean (*n* = 3) ± SD. * *p* < 0.01; *** *p* < 0.0001; ns—non-significant. Full size blots of are shown in Figure S4.

To further explore the role of acetyl-coA elevation in antiandrogen resistance, we examined whether an increase in intracellular acetyl-CoA content could compromise the efficacy of enzalutamide and abiraterone. Until recently, it was believed that acetyl-CoA cannot cross the cell membrane [54]. However, recent studies by Rhee et al. show that exogenous acetyl-CoA can be imported into the cell [55]. The addition of exogenous acetyl-CoA significantly enhanced, in a dose-dependent manner, activation of AR signaling in LNCaP cells stimulated by the synthetic androgen agonist R1881 (Figure 2A,B and Supplementary Figure S2A). Critically, the addition of exogenous acetyl-CoA or its precursor sodium acetate restored AR transcriptional activity in LNCaP cells treated with abiraterone and enzalutamide (Figure 2C and Supplementary Figure S2B). Intra-tumoral androgen biosynthesis has been described as a mechanism of ADT resistance [56]. To determine if the stimulatory effect of acetyl-CoA on AR signaling is a result of increased steroidogenesis, we examined endogenous and secreted testosterone levels in LNCaP and C4-2B cells. As shown in Figure 2D, treatment with acetyl-CoA had no significant impact on the intracellular and secreted testosterone levels in both cell lines. Importantly, treatment with histone acetyltransferase p300/CBP inhibitors A-485 and C646 suppressed AR activity in LNCaP cells cultured with or without supplementation with exogenous acetyl-CoA and reinstated sensitivity to ARSI (Figure 2E and Supplementary Figure S2B). The results of these experiments were further validated with siRNA-mediated knockdowns of p300 and CBP. The expression of PSA, a putative downstream target of AR, was significantly reduced at mRNA and protein levels in LNCaP cells carrying individual or combined knockdowns of CBP and p300 (Figure 3A,B). Taken together, these findings support the critical role of acetylation-dependent mechanisms in conferring antiandrogen resistance.

### 3.3. ACLY Inhibition Results in the Suppression of Ligand-Dependent and -Independent AR Activation

**Figure 4:**
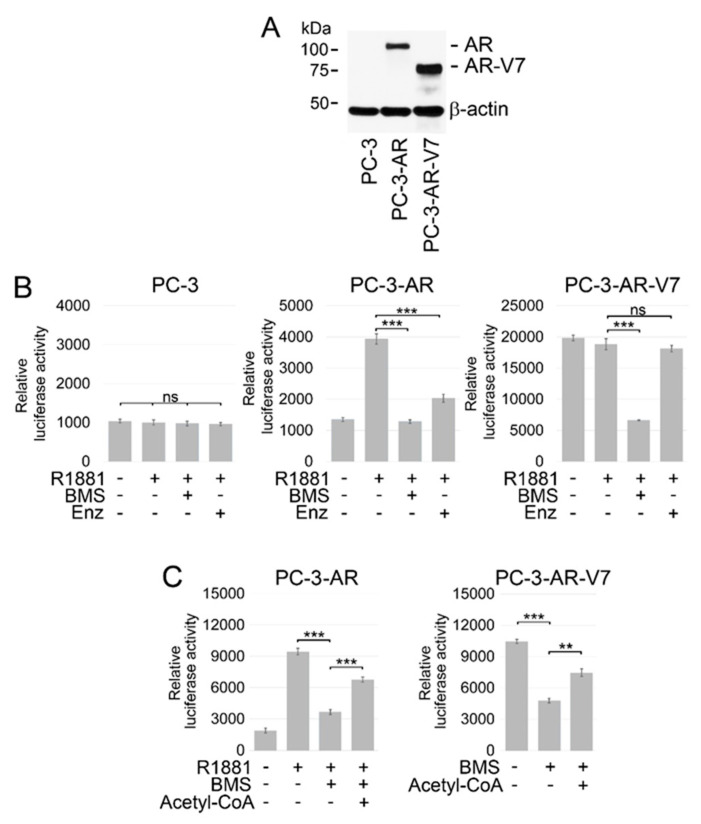
Effect of ACLY inhibition on the transcriptional activity of AR-FL and AR-V7. (**A**) Lysates from parental AR-negative and transformed PC-3 cells expressing the indicated constructs were subjected to Western blotting. (**B**) Parental PC-3 cells or PC-3 cells expressing either full-length AR or AR-V7 were transfected with pGL3-AR-luc and phRL-TK (Renilla) plasmids. The cells were cultured under androgen-depleted conditions (RPMI 1640 medium supplemented with 10% charcoal-stripped FBS) for 24 h followed by treatment with different combinations of BMS 303141 (BMS) (30 μM), enzalutamide (Enz) (2.5 μM), and the synthetic androgen agonist R1881 (1 nM) for 8 h. Samples were assayed for firefly and renilla luciferase activities using the Dual-Glo Luciferase assay. Values were normalized to Renilla activities. (**C**) PC-3 cells expressing either full-length AR or AR-V7 were transfected with pGL3-AR-luc and phRL-TK plasmids. The cells were cultured under androgen-depleted conditions for 24 h followed by treatment with different combinations of BMS 303141 (BMS) (30 μM), acetyl-CoA (0.1 mM), and R1881 (1 nM) for 8 h. Samples were assayed as described in Figure 1B. Columns, means of three different experiments; bars, SDs. ** *p* < 0.001; *** *p* < 0.0001; ns—non-significant. Full size blots of are shown in Figure S4.

Recent studies by Shah et al. show that combined treatment with ACLY and AR inhibitors suppresses AR target gene expression in PC cells expressing AR-FL [57]. However, resistance to ADT and ARSI is attributed, in a large part, to expression of AR-Vs, which facilitate ligand-independent activation of AR signaling [58,59,60,61]. To investigate whether ACLY inhibition affects both ligand-dependent and –independent activation of AR signaling, we stably transfected AR-negative PC-3 PC cells with either AR-FL (PC-3-AR) or AR-V7 (PC-3-AR-V7), the most common constitutively active AR variant expressed in ~70% of CRPC [62] (Figure 4A, Table S1). AR-FL and AR-V7 were expressed in AR-negative PC-3 cells in order to restrict AR signaling to the specific AR isoform. Parental and transformed PC-3 cells were transiently co-transfected with pGL3-ARE-Luc and phRL-TK (Renilla) plasmids. The transfected cells were pre-treated with a potent, cell-permeable ACLY inhibitor BMS 303141 [63,64] and/or enzalutamide followed by stimulation with R1881. As anticipated, luciferase reporter activity was not up-regulated upon treatment with R1881 in reporter-only transfected AR-negative PC-3 cells (Figure 4B). In contrast, R1881 enhanced AR activity in PC-3 cells expressing AR-FL, which was largely suppressed in the presence of BMS 303141 and enzalutamide (Figure 4B). Consistent with the absence of the LBD, AR-V7-mediated reporter activity was not increased upon stimulation of PC-3-AR-V7 cells with R1881. Accordingly, treatment with enzalutamide failed to inhibit AR-reporter activity in these cells. On the contrary, treatment with BMS 303141 resulted in a significant inhibition of AR-reporter activity in PC-3-AR-V7 cells (Figure 4B). To document that the inhibitory effect of BMS 303141 on AR signaling being caused by the inhibition of ACLY-mediated acetyl-CoA synthesis, PC-3-AR, and PC-3-AR-V7 cells transiently co-transfected with pGL3-ARE-Luc and phRL-TK plasmids were treated with BMS 303141 with or without the addition of exogenous acetyl-CoA. As shown in Figure 4C, supplementation with acetyl-CoA restored AR activity in the cells treated with BMS 303141, indicating that the suppressive effect of BMS 303141 on AR signaling was mediated, in a large part, via inhibition of endogenous acetyl-CoA production.

**Figure 5:**
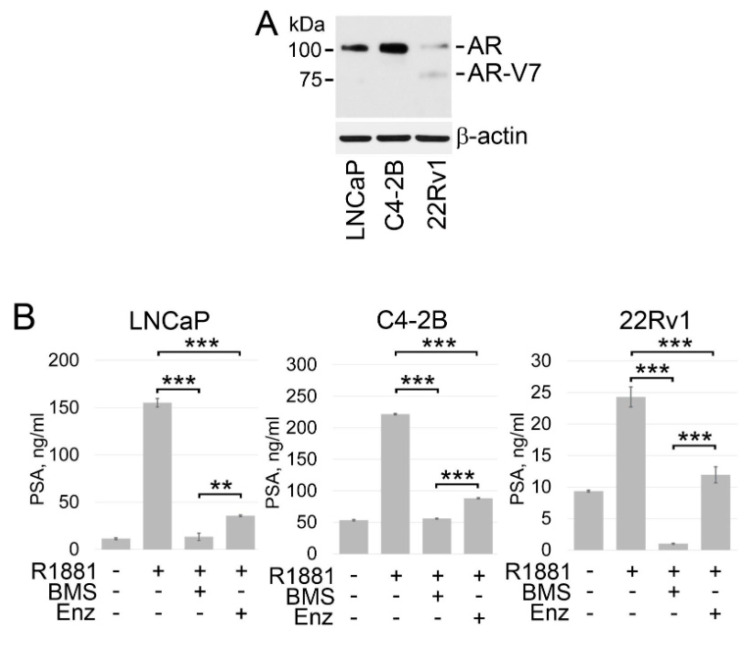
ACLY inhibition results in the suppression of ligand-dependent and -independent PSA expression. (**A**) Western blot analysis of the AR expression in LNCaP, C4-2B, and 22Rv1 PC cells. (**B**) LNCaP, C4-2B, and 22Rv1 cells were cultured under androgen-depleted conditions for 24 h followed by treated with BMS 303141 (BMS) (30 μM), enzalutamide (Enz) (2.5 μM), and R1881 (1 nM) in for 18 hrs. Expression of PSA was examined in the cell culture supernatants using PSA ELISA kit. Columns, means of three different experiments; bars, SDs. ** *p* < 0.001; *** *p* < 0.0001. Full size blots of are shown in Figure S4.

Next, we validated the results of our studies using wild-type AR-positive PC cell lines, specifically androgen-dependent LNCaP and castration-resistant C4-2B and 22Rv1 PC cells. LNCaP and C4-2B cells express functional T877A mutated AR (Figure 5A) [65,66]. The T877A mutation (threonine to alanine substitution at amino acid 877), which resides within the LBD domain of AR, allows the AR to be activated by multiple endogenous hormones and pharmaceuticals [67]. 22Rv1 cells express both full-length H874Y mutated AR and AR-V7 (Figure 5A) [68,69,70,71]. The H874Y mutation within the LBD domain of AR also leads to promiscuous ligand activation [72]. As shown in Figure 5B, treatment with BMS 303141 successfully suppressed PSA expression in all three PC cell lines. Enzalutamide was also able to antagonize R1881-induced expression of PSA in all tested cell lines showing that enzalutamide can achieve on-target inhibition of AR-FL. However, constitutive expression of PSA in 22Rv1 cells was not affected by enzalutamide. These results support the notion that enzalutamide can achieve on-target inhibition of only AR-FL in PC cells, whereas pharmacological inhibition of ACLY may suppress ligand-dependent and –independent activation of AR signaling.

## 4. Discussion

Despite the initial response to androgen blockade, all patients will eventually progress to castration resistance. Castration resistance is progression of disease, either clinical (development of metastatic disease, progression of pre-existing disease) or biochemical (three consecutive rises in PSA levels above nadir) in the presence of castrate levels of circulating testosterone (<20 ng/dL) [73]. Previous studies have identified multiple mechanisms underlying castration resistance, including AR overexpression, extragonadal testosterone synthesis, and acquired sensitivity to non-androgen ligands [22,23,24,25,26,27,28,29,30,31]. Our current findings demonstrate that PC cells respond to androgen ablation by increasing acetyl-CoA expression. Accordingly, the addition of exogenous acetyl-CoA, or its precursor acetate, diminishes the anti-AR activity of abiraterone and enzalutamide. Given that acetylation is an indispensable event for proper AR signaling, these findings suggest that PC cells respond to androgen deprivation by increasing activity of the acetyl-coA pathway in an attempt to restore AR signaling. It is generally assumed that acetyl-CoA cannot cross the cell membrane. However, recent studies by Rhee et al. demonstrated that exogenous supplementation of cell culture media with acetyl-CoA could rescue cell cycle arrest induced by chemical inhibition of ACLY or fatty acid synthase [55]. Furthermore, this study revealed that exogenous ^[3H]^acetyl-CoA could be incorporated into cellular lipids. Rhee et al. suggested that there is a possibility that acetyl-CoA transporter 1 (AT-1), which is expressed on the endoplasmic reticulum (ER) and transports acetyl-CoA into the ER lumen [74], could be exported to the Golgi apparatus, and from the Golgi to the cell membrane [75]. In addition, the acetate moiety from acetyl-CoA could be hydrolyzed from the thioester and provide a source of exogenous acetate. In addition, a possibility exists that acetyl-CoA can enter cells through active phagocytosis or pinocytosis [55].

Our analysis of TCGA dataset shows that only ACLY mRNA expression is significantly increased in tumor tissues compared with normal/benign tissues, whereas ACSS2 mRNA is expressed at the reduced levels in tumors samples (Supplementary Figure S1B). For many gene/protein pairs, the gene expression does not correlate well with the protein expression. Indeed, recent reports show that the expression of both ACLY and ACSS2 is increased at protein levels in prostate cancer tissue [40,76]. A recent report by Shah et al. described a feedback loop between ACLY-dependent fatty acid synthesis and the AR signaling pathway in PC cells [57]. This study suggests that ACLY-dependent fatty acid synthesis plays an important role in sustaining AR action and promoting proliferation of PC cells. Accordingly, ACLY inhibition potentiated the action of enzalutamide in suppressing AR function. Although it has been proposed that the dominant effect of ACLY inhibition observed in this study was due to its impact on fatty acid metabolism, the authors admitted that the role of acetylation could not be ruled out. Indeed, our current study reveals that inhibition of acetyltransferase p300/CBP results in reduced AR transcriptional activity even under supplementation with exogenous acetyl-CoA (Figure 2E). Accordingly, the expression of PSA was significantly reduced at both mRNA and protein levels in LNCaP cells carrying individual or combined knockdowns of CBP and p300 (Figure 3A,B). These findings reinforce the contributing role of acetyl-CoA-dependent acetylation in sustaining AR signaling. Our results are in agreement with previous findings showing that acetylation of AR is required for its transcriptional activity [41,43,44,45] and protects the AR protein from polyubiquitination and proteasomal degradation [77]. Recent studies by Welti et al. demonstrate that CCS1477, an orally bioavailable and selective inhibitor of the p300/CBP, potently blocks both AR-FL and AR-V7 signaling and exhibits prominent antitumor activity in cell and animal models of human prostate cancer [78].

Intra-tumoral androgen biosynthesis has been described as one of a key mechanisms of ADT resistance [75]. Studies by Zadra et al. reveled that following blockade of fatty acid synthesis and unused acetyl-CoA may be redirected toward the cholesterol pathway [79]. Therefore, the possibility exists that acetyl-CoA may potentially compromise the efficacy of antiandrogens by serving as a precursor for cholesterol synthesis and finally testosterone production. Yet, the supplementation with exogenous acetyl-CoA did not produce any significant impact on the intracellular and secreted testosterone levels in PC cells (Figure 2D). These findings support previous observation that increase in acetyl-CoA levels does not lead to enhanced steroidogenesis in PC cells [67]. Studies by Yu et al. indicate that organelle-derived acetyl-CoA may support pro-tumorigenic properties of prostate cancer cells, i.e., survival, migration, and metastasis, via activation of calmodulin kinase II [80]. Yet, the authors admit that the involvement of cytosolic-generated acetyl-CoA in CaMKII activation cannot be excluded.

## 5. Conclusions

Our current studies demonstrate that treatment with enzalutamide and abiraterone elevates intracellular levels of acetyl-CoA in PC cells. Accordingly, the addition of exogenous acetyl-CoA compromises the anti-AR activity of these antiandrogens. Our experiments also established the critical role of the acetyl-CoA pathway for both ligand-dependent and -independent routes of AR activation. Therefore, aberrant acetyl-CoA homeostasis may play a critical role in the development of primary and acquired resistance to therapeutics targeting the AR signaling axis (Supplementary Figure S3).

## Data Availability

The data presented in this study are available from the corresponding author (V.M.K.) upon request.

## Supplementary Materials

The following supporting information can be downloaded at: https://www.mdpi.com/article/10.3390/cancers14235900/s1, Figure S1: Analysis of ACLY and ACSS2 expression in normal and cancerous prostate tissue. Figure S2: Acetyl-CoA diminishes the inhibitory effect of abiraterone and enzalutamide on AR signaling. Figure S3: Aberrant acetyl-CoA homeostasis promotes resistance to antiandrogen therapeutics. Figure S4: Full size blots of Figure 3A, Figure 4A, and Figure 5A. Table S1: Primer sequences for amplification of the indicated variants of AR.
