# Supplementary material for: Acetyl-CoA Counteracts the Inhibitory Effect of Antiandrogens on Androgen Receptor Signaling in Prostate Cancer Cells
Source: Cancers (Basel). 2022 Nov 29;14(23):5900. doi: 10.3390/cancers14235900 (PMC9738902; doi:10.3390/cancers14235900)
Supplement: Supplementary file 1 [file cancers-14-05900-s001.zip › cancers-2027226-supplementary.pdf]

Table S1. Primer sequences for amplification of the indicated variants of AR. Restriction sites are underlined.

AR full	Forward	5′-atcttt tctaga atggaagtgcagttagggctgg
	Reverse	5′-atcttt ctcgag ttatataacaggcagaagacatctgg
AR-V7	Forward	5′-atcttt gaattc atggaagtgcagttagggctgg
	Reverse	5′-atcttt ggatcc tcagggtctggtcattttgagatgc

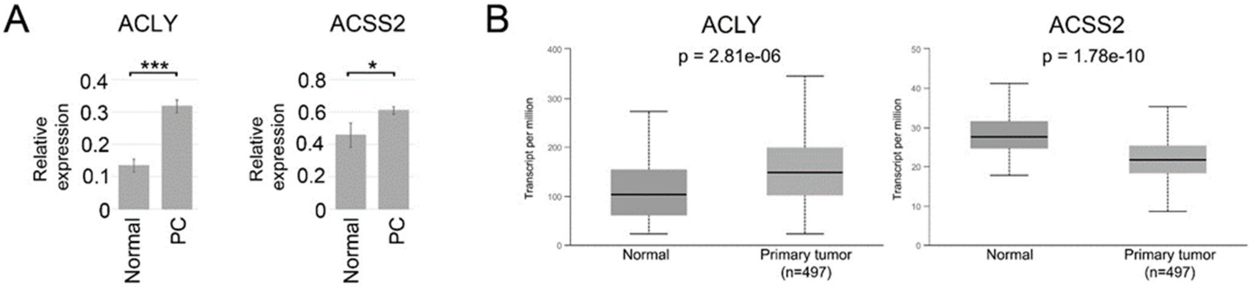

Figure S1. Analysis of ACLY and ACSS2 expression in normal and cancerous prostate tissue. (a) Quantification of tissue microarray (TMA) staining for ACLY (normal prostate (n = 12), PC (n = 39)) and ACSS2 (normal prostate (n = 5), PC (n = 21)). Staining and evaluation of TMA was performed at the Fox Chase Cancer Center Histopathology Facility using Ventana Discovery Ultra platform and Leica Aperio system. Results are expressed as the mean (n=3)±SEM. *P<0.05; ***P<0.0001. (b) TCGA analysis of ACLY and ACSS2 expression in normal and cancerous prostate tissue. TCGA dataset analysis was performed via the University of ALabama at Birmingham CANcer data analysis Portal (UALCAN).

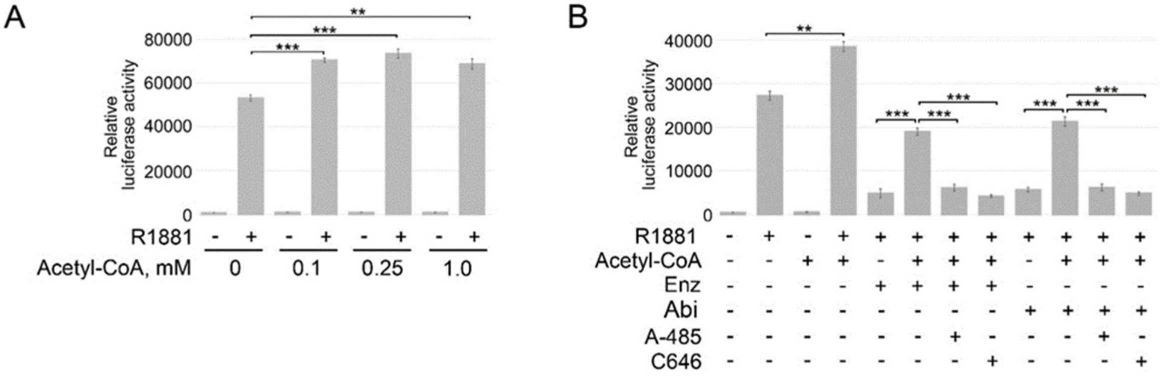

Figure S2. Acetyl-CoA diminishes the inhibitory effect of abiraterone and enzalutamide on AR signaling. (a) LNCaP cells with stable expression of AR luciferase reporter were cultured under androgen-depleted conditions for 24 hours followed by treatment with R1881 (1nM) and the indicated concentrations of acetyl-CoA for 16 hrs. (b) LNCaP cells with stable expression of AR luciferase reporter were cultured under androgen-depleted conditions for 24 hours followed by treatment with enzalutamide (Enz) (1◎M), abiraterone acetate (5◎M) (Abi), R1881 (1nM), and acetyl-CoA (0.1mM) for 16 hrs. Samples were assayed for firefly luciferase activity using the Dual-Glo Luciferase assay. Results are expressed as the mean (n=3)±SD. **P<0.001; ***P<0.0001.

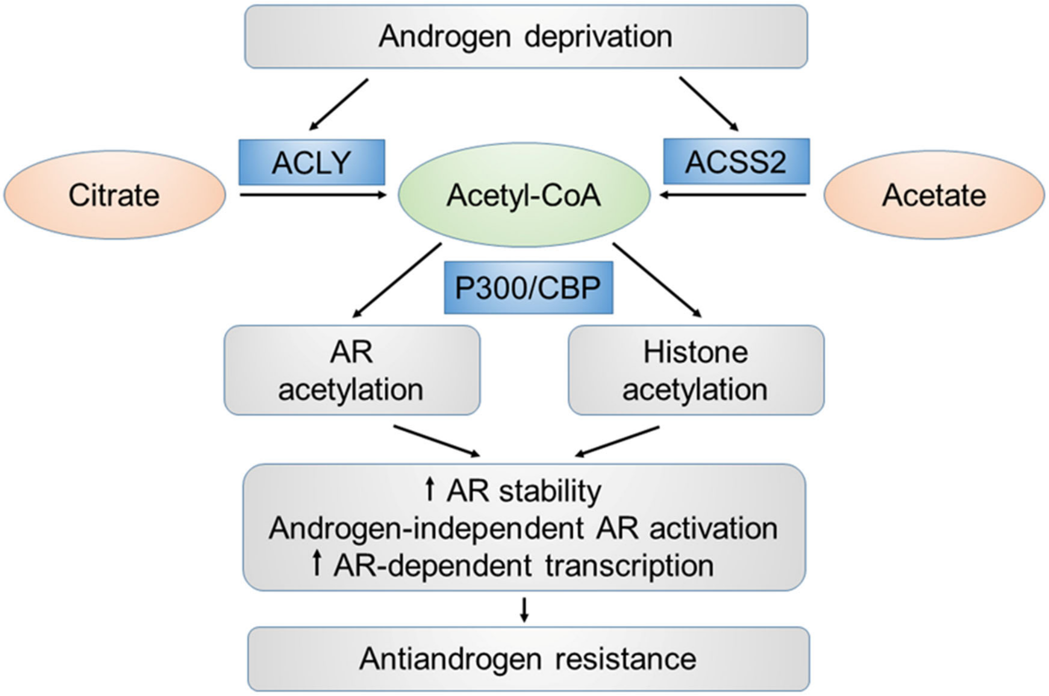

Figure S3. Aberrant acetyl-CoA homeostasis promotes resistance to antiandrogen therapeutics.

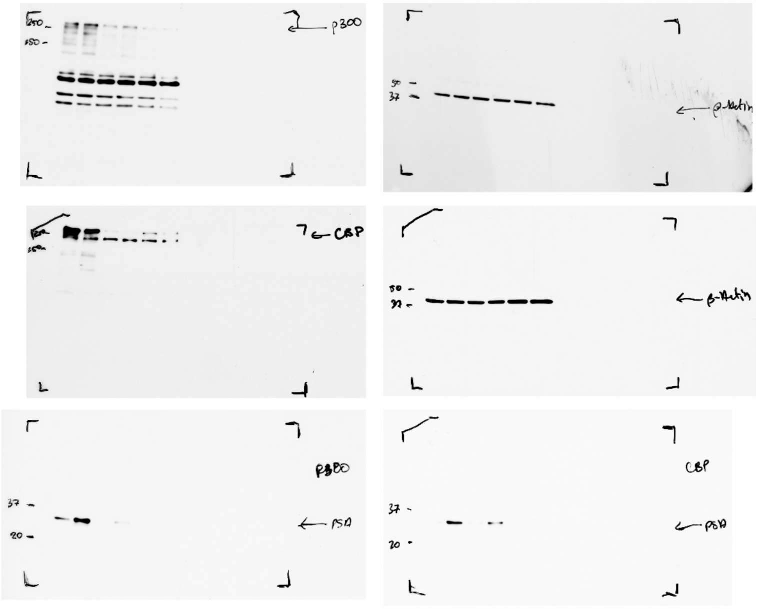

Full size blots of Figure 3A.

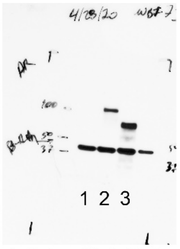

Full size blot of Figure 4A.
Lane 1 - PC-3 cells
Lane 2 - PC-3-AR cells
Lane 3 - PC-3-AR-V7 cells

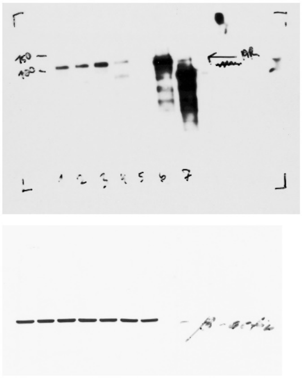

Full size blot of Figure 5A.
Lane 2 - LNCaP cells
Lane 3 - C4-2B cells
Lane 4 - 22Rv1 cells

Figure S4. Full size Western blots.
